# Human Leukocyte Antigen–Haploidentical Haematopoietic Stem Cell Transplantation Using Post-Transplant Cyclophosphamide for Paediatric Haematological Malignancies

**DOI:** 10.3390/cancers16030600

**Published:** 2024-01-31

**Authors:** Takuro Nishikawa

**Affiliations:** Department of Pediatrics, Graduate School of Medical and Dental Sciences, Kagoshima University, Kagoshima 890-8520, Japan; adu44150@ams.odn.ne.jp; Tel.: +81-99-275-5354

**Keywords:** children, haematopoietic stem cell transplantation, human leukocyte antigen–haploidentical, post-transplant cyclophosphamide, haematological malignancies

## Abstract

**Simple Summary:**

Human leukocyte antigen (HLA)–haploidentical haematopoietic stem cell transplantation (haplo-HSCT) with post-transplant cyclophosphamide (PTCY) for haematological malignancies is an HSCT modality that has become widespread worldwide in the last 20 years and is also widely used in children. Haplo-HSCT with PTCY in adult patients with haematologic malignancies has comparable outcomes with HLA-matched unrelated donor HSCT. This review article comprehensively describes the current progress in haplo-HSCT with PTCY for paediatric haematological malignancies. The current state and future directions for donor selection (sex, age, ABO blood type, and HLA disparity), donor source, the dose of infused CD34^+^ cells, optimal conditioning, and the concomitant graft-versus-host disease prophylaxis other than PTCY are also extensively discussed. These aspects present key solutions for further improvements in the outcomes of haplo-HSCT with PTCY for paediatric haematological malignancies.

**Abstract:**

The use of human leukocyte antigen (HLA)–haploidentical haematopoietic stem cell transplantation (HSCT) with post-transplant cyclophosphamide (PTCY), which markedly reduces the risk of graft-versus-host disease, has rapidly increased worldwide, even in children. It was initially developed for post-transplant relapse or non-remission at transplant for patients with high-risk haematologic malignancies. However, this strategy is currently used more frequently for standard-risk, transplant-eligible paediatric haematological malignancies. It has recently been recognised in adults that the transplant outcomes after PTCY-based HLA–haploidentical HSCT are comparable with those achieved after HLA-matched HSCT. Therefore, even in children, parental donors who are HLA–haploidentical donors and cord blood are currently considered the next donor candidates when an HLA-matched related or unrelated donor is unavailable. This review addresses the current status of the use of haplo-HSCT with PTCY for paediatric haematologic malignancies and future directions for donor selection (sex, age, ABO blood type, and HLA disparity), donor source, the dose of infused CD34^+^ cells, optimal conditioning, the concomitant graft-versus-host disease prophylaxis other than PTCY, and the pharmacokinetic study of CY and CY metabolites. These aspects present key solutions for further improvements in the outcomes of haplo-HSCT with PTCY for paediatric haematological malignancies.

## 1. Introduction

It has been 20 years since O’Donnell et al. of Johns Hopkins University first reported on human leukocyte antigen (HLA)–haploidentical haematopoietic stem cell transplantation (HSCT) with post-transplant cyclophosphamide (PTCY) [1].

PTCY is an inexpensive transplantation method with excellent graft-versus-host disease (GVHD) suppression efficacy; therefore, it is popularly used globally with innovations such as the use of peripheral blood stem cells, bone marrow, and enhanced conditioning regimens. In Japan, the number of haplo-HSCTs in 2020 exceeded that of HLA-matched related donor HSCTs [2]. Notably, 90% of haplo-HSCTs in the United States are performed with PTCY-based GVHD prophylaxis [3]. In adults, a small number of prospective randomised trials and a large number of retrospective studies have shown that the results from using haplo-HSCT with PTCY in patients with haematologic malignancies are comparable with those of HLA-matched unrelated HSCTs [4,5,6].

Haplo-HSCT with PTCY for paediatric haematologic malignancies was initially performed with the expectation of its anti-tumour effects in refractory cases, such as non-remission after intensive chemotherapy or relapse after HSCT. Following reports in adults, haplo-HSCT with PTCY is increasingly being used as an alternative donor in the absence of an HLA-matched donor, even during remission. This article discusses the current status of the use of haplo-HSCT with PTCY for paediatric haematologic malignancies. The review also addresses donor selection (sex, age, HLA disparity, and ABO blood type), donor source, the dose of infused CD34^+^ cells, optimal conditioning, and the concomitant GVHD prophylaxis other than PTCY. This may affect the outcome of haplo-HSCT with PTCY. Furthermore, the differences between this regimen and another typical haplo-HSCT regimen that uses anti-thymocyte globulin (ATG)-based GVHD prophylaxis have been highlighted [7,8].

## 2. Basic Theory of PTCY

PTCY has a long history of use. In 1963, a mouse model of major histocompatibility complex (MHC)-incompatible skin grafts showed that the anti-rejection effect of cyclophosphamide (CY) was dependent on the timing of CY administration and was highest when CY was administered 1–4 days after the graft [9]. It also showed that the immune tolerance effect of CY was cancelled by the concomitant use of calcineurin inhibitors [10]. This was assumed to be because calcineurin inhibitors suppress the activation of alloreactive T cells and reduce their sensitivity to CY [10]. Based on the results of these basic studies, the Johns Hopkins group decided that the use of immunosuppressive drugs, such as calcineurin inhibitors and steroids, should be avoided immediately after graft infusion until after PTCY administration [1].

**Figure 1:**
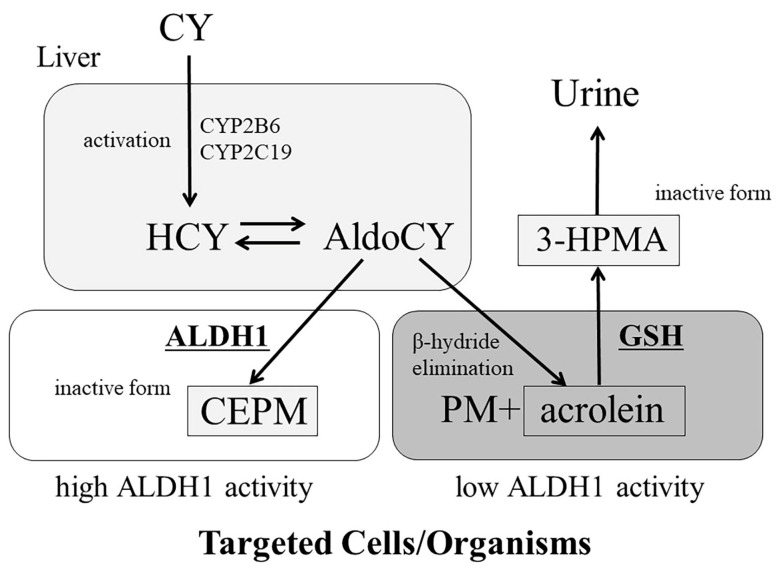
Cyclophosphamide metabolic pathways.

CY is catalysed by the cytochrome P-450 enzyme in the liver (CYP2B6, CYP2C19, etc.) and is activated by 4-hydroxycyclophosphamide (HCY) and its tautomer aldocyclophosphamide (AldoCY) (Figure 1). AldoCY diffuses and circulates passively from hepatocytes and undergoes a beta-elimination reaction in each cell, exhibiting alkylating activity as phosphoramide mustard (PM). During this process, acrolein is formed as a side reaction product [11]. In contrast, high intracellular aldehyde dehydrogenase 1 (ALDH1) activity irreversibly converts AldoCY to the inactive non-cytotoxic metabolite carboxyethylphosphoramide mustard (CEPM). Therefore, haematopoietic stem cells and regulatory T cells with high ALDH1 expressions are less susceptible to CY-induced damage [12,13,14]. Regulatory T cells regulate GVHD; this is one of the reasons PTCY can control GVHD [12,15]. Acrolein, which is produced during the metabolic conversion of CY to PM, has long been known to cause haemorrhagic cystitis in patients using CY and has recently attracted attention as a cause of CY-induced cardiotoxicity [13,14]. CY is highly toxic to rapidly dividing cells; therefore, it is used as an anticancer agent, primarily for haematologic malignancies. It is also widely used to treat autoimmune diseases because of its immunosuppressive effects owing to its strong action towards lymphocytes [16]. In 2001, a group from Johns Hopkins University reported that HLA–haploidentical bone marrow transplantation (BMT) with PTCY was established in a mouse model [17]. This report presented, for the first time, a prototype of the current haplo-BMT with PTCY using reduced intensity conditioning (RIC) consisting of fludarabine, total body irradiation (TBI) (2 Gy), and CY on day 3 after transplantation. In a mouse model, Ross et al. confirmed that T cells responding to allogeneic antigens are more sensitive to PTCY than those not responding to allogeneic antigens [18]. In addition, several mechanisms underlying the GVHD-suppressing effects of PTCY have recently been reported. PTCY reduces alloreactive CD4^+^ effector T cell proliferation and induces the dysfunction of alloreactive CD4^+^ and CD8^+^ effector T cells [19,20]. Furthermore, PTCY increases the number of myeloid-derived suppressor cells and preferentially enhances the recovery of CD4^+^ regulatory T cells [21].

Cyclophosphamide (CY) is metabolised to 4-hydroxycyclophosphamide (HCY) in the hepatic cytochrome P-450 enzyme (CYP) system (CYP2B6 and/or CYP2C19). HCY enters cells as the tautomer aldocyclophosphamide (AldoCY). AldoCY can be converted to phosphoramide mustard (PM) and acrolein via β-elimination. Alternatively, aldehyde dehydrogenase 1 (ALDH1) oxidises AldoCY to the inactive metabolite o-carboxyethylphosphoramide mustard (CEPM). Glutathione (GSH) metabolises acrolein to the inactive metabolite 3-hydroxypropyl mercapturic acid (3-HPMA).

## 3. Development of Haplo-HSCT with PTCY and Its Current Progress in Adults

O’Donnell et al. were the first to report HLA–haplo-BMT with PTCY at Johns Hopkins in 2002 [1]. The conditioning regimen comprised fludarabine (150 mg/m^2^) + TBI (2 Gy). GVHD prophylaxis comprised a single dose of CY (50 mg/kg) on day 3 post-transplant, and tacrolimus and mycophenolate mofetil (MMF) started from day 4. As two of the three initial patients developed graft failure, CY (14.5 mg/kg × 2 days) was added to the conditioning regimen, and engraftment was observed in 8 of the 10 patients. This revealed that in haplo-HSCTs with PTCY, it is necessary to suppress host lymphocytes and the host-versus-graft response using a certain level of conditioning treatment. In a report of 68 patients who used this conditioning regimen, the 1-day (day 3) versus 2-day (days 3 and 4) administration of PTCY was compared. Engraftment was achieved in 87% of patients, 34% of whom had acute GVHD of the II–IV degree and 6% had acute GVHD of the III–IV degree. Safety was excellent, with a 1-year non-relapse mortality rate of 15%. However, the relapse rate was high at 51%; overall survival at 2 years was 36%, and event-free survival at 2 years was 26%. There was no difference in the survival or incidence of acute GVHD according to the number of days of PTCY administration. However, the incidence of systemic chronic GVHD was 25% in the 1-day group and only 5% in the 2-day group. Based on these results, a 2-day administration of PTCY has become the standard strategy [22,23].

Raiola et al. reported haplo-BMT with PTCY using myeloablative conditioning (MAC) for adult haematologic malignancies (54% non-remission) [24]. The conditioning regimen comprised fludarabine (120 mg/m^2^) + TBI (9.9–12 Gy) or thiotepa (5–10 mg/kg) + busulfan (9.6 mg/kg) + fludarabine (150 mg/m^2^). The relapse rate at 6 months after haplo-BMT was 22%, non-relapse mortality was 18%, overall survival at 18 months was 62%, and disease-free survival was 51%, indicating that MAC may reduce the relapse rate and improve transplant outcomes in indicated cases. In addition, haplo-peripheral blood stem cell transplantation (PBSCT) with PTCY was developed to improve the engraftment rate and enhance the graft-versus-leukaemia effect [25,26,27]. Raj et al. performed haplo-PBSCT with PTCY at four centres and retrospectively analysed 55 cases [25]. Except for the use of PBSC instead of BM, the Johns Hopkins method was used for the conditioning regimen and the prophylaxis of GVHD, with 96% of the patients achieving engraftment, 51% developing grade II acute GVHD, 8% developing grade III GVHD, and 18% developing chronic GVHD at 2 years. The overall survival at 2 years was 48%, and the event-free survival was 51%. Compared with the BMT report, there was a slight increase in acute GVHD incidence, whereas the recurrence rate at 2 years was somewhat lower at 28%. Haplo-HSCT with PTCY was initially developed as a BMT after RIC; however, it is also used in PBSCT and MAC. In adults, as with other HLA-matched allogeneic HSCTs, the donor source and conditioning regimen are selected on a case-by-case basis.

A retrospective study was conducted using the Center for International Blood and Marrow Transplant Research (CIBMTR) patient registry to compare the outcomes of haplo-HSCT with PTCY and other transplantation methods in adult patients with acute lymphoblastic leukaemia (ALL) in remission (4201 patients) [6]. In comparing the outcomes of haplo-HSCT with PTCY and HSCT from a matched sibling donor (MSD), no differences were noted in survival, leukaemia-free survival, non-relapse mortality, relapse rate, or the occurrence of acute GVHD; however, the occurrence of chronic GVHD was higher in the MSD group than in other groups. When comparing the outcomes of haplo-HSCT with PTCY and HSCT from a matched unrelated donor (MUD), no difference in survival, non-leukaemia-free survival, or relapse rate was observed. However, non-relapse mortality, the occurrence of grade III–IV acute GVHD, and the occurrence of chronic GVHD increased in the MUD group. HSCT from a mismatched unrelated (MMUD) donor or cord blood transplantations (CBTs) also showed decreased survival and increased non-relapse mortality and the occurrence of grade III–IV acute GVHD compared with haplo-HSCT with PTCY. Therefore, haplo-HSCT with PTCY is the preferred alternative transplantation method in adult patients with ALL.

## 4. Haplo-HSCT with PTCY and Its Current Progress in Paediatric Patients

The reports on haplo-HSCT with PTCY in children are summarised in Table 1 [28,29,30,31,32,33,34,35,36]. Notably, most paediatric reports are single-centre studies, analyse a small number of patients, include mixed underlying diseases, and are retrospective studies. However, these reports show that haplo-HSCT with PTCY is feasible, as in adults, whether the donor source is PBSC or BM and whether the conditioning regimen is MAC or RIC.

Saglio et al. retrospectively compared 23 patients who received haplo-HSCT with PTCY for childhood acute leukaemia with 41 patients who received HSCT from MUDs and 26 patients who received HSCT from MMUDs and found no difference in the 5-year overall survival rates [35]. 

In addition, a report from Brazil of a relatively large retrospective study of haplo-HSCT with PTCY in 144 children with acute leukaemia (median age 10 years; 86 with ALL and 58 with acute myeloid leukaemia (AML); 40 in first remission, 57 in second remission, 27 in third remission or higher, and 20 in non-remission) reported that the 2-year overall survival, leukaemia-free survival, and graft-versus-host relapse-free survival (GRFS) were 52%, 44%, and 34%, respectively [36]. Furthermore, the cumulative incidence of chronic GVHD and relapse rate at 2 years were 31% and 40%, respectively. The acute leukaemia status at the time of HSCT was associated with the prognosis. The prognosis was better if the patient was in remission at the time of HSCT, with an even lower risk of relapse if minimal residual disease was negative. Notably, no large prospective studies have been conducted; however, haplo-HSCT with PTCY may be an appropriate clinical option for children without MSD or MUD donors who require HSCT. 

At our centre, the choice of haematopoietic stem cell source in haplo-HSCT with PTCY for paediatric haematological malignancy is in principle PB. The reason for this is that GVHD has a low risk to some extent in this HSCT setting and can elicit an anti-tumour effect. For paediatric non-malignancy, BM is selected. The reason for this is that the anti-tumour effect is not necessary and the risk of chronic GVHD is reduced as much as possible. Notably, various conditioning regimens have been reported in paediatric settings, from RIC to MAC [28,29,30,31,32,33,34,35,36], and the choice of conditioning regimen should be based on the disease status and comorbidities. The conditioning regimen for paediatric haematological malignancy is a TBI-based regimen for ALL and either a TBI-based or a non-TBI (melphalan or busulfan)-based regimen for AML [24]. For a second HSCT, non-TBI is chosen if TBI was performed during the first HSCT. If the patient has organ complications, an RIC regimen is an option [22]. However, if the patient is in non-CR or minimal/measurable residual disease (MRD) positive for the HSCT, an enhanced conditioning regimen is preferable as far as possible. Specifically, at our centre, the conditioning regimens used are shown in Table 2. The TBF regimen (thiotepa 10 mg/kg, busulfan: 9.6 mg/kg, and fludarabine 150 mg/m^2^) is one of the most commonly used MAC regimens worldwide and is also used in children [36]. However, thiotepa cannot be used in combination with busulfan in Japan; therefore, the TBF regimen is not used at our centre. 

In the absence of HLA-matched related/unrelated donors, related haploidentical donors or umbilical cord blood donors are candidates. Both donors allow for rapid donor availability and flexibility in the timing of HSCT. Several comparative studies have compared haplo-HSCT with PTCY and CBT for haematological malignancies, primarily in adults [4,37,38,39,40]. No significant difference was noted in disease-free survival between the two transplantation method groups, whether studied in RIC [4,37] or MAC [38]. In the United States, the BMT CTN 1101 trial, a phase III randomised trial of double CBT and haplo-BMT with PTCY in patients with acute leukaemia and lymphoma in remission, was conducted [4]. The primary endpoint of 2-year progression-free survival was not statistically different. However, the 2-year non-relapse mortality of 18% for CBT was significantly higher than the 11% rate found for haplo-HSCT with PTCY (*p* = 0.04), and the 2-year overall survival rate of CBT was also inferior to that of haplo-HSCT with PTCY (46% vs. 57%, *p* = 0.04). In the report by Wagner et al. [38], only a paediatric cohort (<16 years) was examined, and still no significant difference in leukaemia-free survival by donor type (haplo-donor or UCB) was found. However, in a comparative study of haplo-HSCT with PTCY and UCB for adult lymphoma, progression-free survival and overall survival were significantly longer after haplo-HSCT [39]. Although a prospective randomised study of adult haematological malignancies under MAC conditions was also conducted, the study was terminated mid-study because of poor recruitment. Owing to the smaller number of patients enrolled, adequate statistical detection was not available; nevertheless, the GRFS of haplo-HSCT with PTCY was substantially better than that of CBT (no difference in event-free survival and overall survival) [40]. Based on the above, I believe that at present, in the absence of an HLA-matched related/unrelated donor, haplo-HSCT with PTCY rather than CBT is recommended for patients with paediatric haematological malignancies.

## 5. Measures to Improve the Outcomes of Paediatric Haplo-HSCT with PTCY

The following sections discuss donor selection, donor source, the number of infused CD34^+^ cells, the optimal conditioning regimen, and the concomitant GVHD prophylaxis other than PTCY as factors that can be adjusted to improve the outcomes of haplo-HSCT with PTCY.

For donor selection, The European Society for Blood and Marrow Transplantation (EBMT) has published the following consensus recommendations: [41] (a) for a recipient with donor-specific anti-HLA antibodies, a donor without the corresponding HLA antigen is preferred (mean fluorescence intensity (MFI) < 1000); (b) a younger donor is preferred to an older donor; (c) a male donor is preferred to a male recipient; and (d) a sibling or offspring donor is preferred to a parent donor. Between parent donors, a father donor is preferred to a mother donor. An ABO-matched donor is preferred to a minor ABO-mismatched donor, and a minor ABO-mismatched donor is preferred to a major ABO. Notably, the cut-off MFI values may vary among laboratories. Although these recommendations are not explicitly tailored for children, they are considered equally applicable in paediatric cases. In a retrospective study of paediatric haplo-HSCT with PTCY, Rocha et al. found that maternal donors were associated with an increased risk of chronic GVHD and decreased overall survival and GRFS [36]. With regard to HLA mismatches, a class II HLA mismatch may improve the outcomes following haplo-HSCT with PTCY [42,43]. A single class II HLA mismatch, e.g., an HLA-DR, HLA-DQ, or HLA-DP mismatch, had a protective effect on disease-free and overall survival, primarily as a result of reduced relapse risk in a single-centre study with adult patients [42]. Furthermore, this survival effect was cumulative; thus, patients with three class II mismatches had the best overall survival [42].

Regarding stem cell sources, the data are mostly from adults; however, a meta-analysis reported an increase in chronic GVHD occurrence or a lower recurrence rate in PBSC than in BM [44]. The analysis included non-neoplastic diseases in the U.S.; however, PB accounts for approximately 80% of patients aged >18 years and approximately 40% of patients aged <18 years, with PB being the less common choice in children [3]. BM is likely to be selected more often in non-neoplastic diseases; therefore, disease-specific analyses are required. This choice of stem cell sources should be based on individual cases, considering the risk of chronic GVHD and relapse.

Regarding the dose of infused cells, using GVHD prophylaxis with ATG for 29% and PTCY for the remaining 71%, a retrospective study of EBMT of haplo-PBSCT reported a CD34^+^ cell count of more than 4.96 × 10^6^/kg was correlated with more significantly decreased non-relapse mortality, prolonged leukaemia-free survival, and overall survival [45]. A single-centre retrospective study of haplo-PBSCT with PTCY also analysed the outcomes by stratifying the infusion of CD34^+^ cells into low- (<5 × 10^6^/kg), intermediate- (5–10 × 10^6^/kg), and high-dose (>10 × 10^6^/kg) groups. In the multivariate analysis, the low-dose CD34^+^ cell group had worse non-relapse mortality, progression-free survival, and overall survival than that the intermediate group. Clinical outcomes in the intermediate- and high-dose CD34^+^ transplant groups were similar, and the dose of CD3^+^ cells had no significant effect on outcome; therefore, a CD34^+^ cell count greater than 5 × 10^6^/kg may improve survival [46]. It has also been reported that the clinical effects of infused CD34^+^ cell doses occur only in HSCTs from haploidentical donors [47]. There are a few reports on the dose of infused CD34^+^ cells in paediatric haplo-HSCT with PTCY. In paediatric cases, the donor is often the parent, and the dose of infused cells per kilogram of the recipient’s body weight is often high, owing to the weight difference between the recipient and donor. Therefore, it is unknown whether infusing large doses of cells, such as more than 20 × 10^6^/kg CD34^+^, is acceptable. There is concern that the incidence of GVHD may increase. 

Regarding combined GVHD prophylaxis other than PTCY, the Johns Hopkins method of initiating calcineurin inhibitors (tacrolimus or cyclosporine) and MMF on day 5 after PTCY in children has been widely reported [28,29,30,31,32,33,34,35,36]. Recently, the timing of calcineurin inhibitor initiation has been discussed. In paediatric haplo-HSCT with PTCY, cytokine release syndrome was less likely to occur when calcineurin inhibitors were administered before PTCY [48]. In addition, a retrospective study in adults reported the possibility of improving leukaemia-free survival and GRFS by administering calcineurin inhibitors and MMF on day 0 or day 1 [49]. However, a comprehensive gene expression analysis of donor T cells in a mouse model recently showed that GVHD prophylaxis with cyclosporine inhibited donor T cell exhaustion and induced highly active transitory exhausted T cells (transitory-Tex), leading to the development of chronic GVHD [50]. Additionally, the early initiation of calcineurin inhibitors before PTCY induced transitory-Tex and inhibited tolerance induction after haplo-PBSCT with PTCY [51]. Consequently, the administration of a calcineurin inhibitor after PTCY is essential for tolerance induction without chronic GVHD. Therefore, continued careful discussion regarding the early initiation of calcineurin inhibitors is necessary.

The disease status at HSCT is also a factor that significantly influences transplant outcomes [36]. The use of novel drugs, such as blinatumomab [52], inotuzumab ozogamicin [53], and gilteritinib [54], before transplantation with the aim of achieving an MRD-negative status in HSCT could also be an essential strategy. In addition, haplo-HSCT with PTCY is likely to allow the initiation of maintenance therapies, such as molecular-targeted drugs and immunotherapy, soon after HSCT because of favourable engraftment, good blood cell recovery, low GVHD risk, low risk of complications, including infection, and the possibility of early discontinuation of immunosuppressive drugs. In the future, post-HSCT maintenance therapy will be increasingly developed to treat haematologic malignancies [55,56,57], and haplo-HSCT with PTCY, compatible with the maintenance therapies described above, will be a valuable method in HSCT.

Haplo-HSCT with high-dose ATG and strengthened immune suppression with G-CSF-mobilised grafts (BM and PB), as represented by the Beijing protocol, was compared with haplo-HSCT with PTCY in a paediatric setting. The relatively new Beijing protocol has been reported to have high overall and leukaemia-free survival rates, with both parameters increasing to approximately 80% [58,59,60], compensating for a significantly higher incidence of acute and chronic GVHD. The difference between the Beijing protocol and haplo-HSCT with PTCY was that the risk of GVHD was higher in the former. Therefore, avoiding chronic GVHD, especially in children, is a major consideration when selecting an HSCT procedure, and long-term quality of life must be maintained.

## 6. Viral Reactivation and Haemorrhagic Cystitis in Paediatric Haplo-HSCT with PTCY

Patients receiving HSCT from haplo-donors are likely to be more severely immunocompromised than those receiving HSCT from matched donors [61]. The pattern of immune reconstitution in patients after haplo-HSCT with PTCY was reported to be more along the lines of impaired T cell and natural killer cell recovery than in patients after HSCT from a matched donor [62]. The use of PTCY itself is also associated with increased cytomegalovirus (CMV) infection [63]. The incidence of CMV infection after haplo-HSCT with PTCY is 30–54% [61,62,63,64,65]. Furthermore, patients with CMV reactivation in this HSCT setting have a higher cumulative incidence of non-relapse mortality than those patients without CMV reactivation [65]. If the patient’s serum CMV is positive, the physician should be cautious because of the high risk of CMV reactivation [63,65]. Based on the above, an active prophylaxis strategy for CMV reactivation is supported in all patients undergoing haplo-SCT with PTCY. The novel CMV DNA terminase complex inhibitor, letermovir, was also effective in preventing CMV infection among patients receiving haplo-HSCT with PTCY [66]. Non-CMV herpes viruses are also at high risk of reactivation in haplo-HSCT with PTCY, especially human herpesvirus 6. Non-CMV herpesvirus infection is also still associated with increased post-transplant non-relapse mortality [67]. Nevertheless, several reports exist that demonstrate less reactivation of CMV infection with PTCY than with ATG in haplo-HSCT [64,68]. As well as viral infections, HSCT with PTCY is a risk factor for the development of bacterial [69] and fungal infections [70], regardless of the donor, and both infections are associated with increased mortality.

Haemorrhagic cystitis (HC) is one of the relatively frequent complications after allogeneic HSCT. HC is believed to be caused by the interaction of diverse factors, including conditioning drugs, immunosuppressive drugs, donor–recipient HLA mismatch, and viral reactivation. Moreover, using CY before or after HSCT, or the toxic metabolite of CY, acrolein, could damage the urothelium and be the first hit in the multi-step pathogenesis process of HC [71,72,73]. The most common viruses associated with HC are BK virus (BKV) and adenovirus (AdV), both of which are latently infectious after initial infection and reactivated by highly suppressed cellular immunity, causing HC [71,72,73]. In Europe and the USA, most HCs are attributed to BKV, whereas in Japan, approximately half of all HCs are attributed to AdV type 11 [74]. This difference may be due to the high rate of previous AdV type 11 infection in the Japanese population [74]. In an adult retrospective study, the incidence of HC 1 year after HSCT was markedly higher in haplo-HSCT with PTCY, at 46.0% compared with 25.8% in HSCT from MRD [75]. HC might be a risk factor for transplantation-related mortality [73]. Therefore, in haplo-HSCT with PTCy, this complication is of great concern. In HC involving BKV and AdV, although cidofovir is commonly used, it is not sufficient in terms of efficacy [76]. Therefore, the development of methods to prevent or treat HC is desirable. A recent report showed that continuous rather than intermittent bolus administration of mesna (sodium salt of 2-mercaptoethanesulfonate) detoxified acrolein and reduced the incidence of HC after haplo-HSCT with PTCY, probably because it prevented bladder and urinary tract damage due to CY [77].

## 7. Pharmacokinetic (PK) Study of CY and CY Metabolites and Cardiotoxicity in Paediatric Haplo-HSCT with PTCY

Regarding the PK study of CY and CY metabolite concentrations (HCY and CEPM) at high-dose CY (60 mg/kg × 2) regimens in conditioning and post-transplant outcomes, the incidence of sinusoidal obstruction syndrome/veno-occlusive disease after HSCT was higher in the group with a higher area under the concentration–time curve (AUC) of CEPM [11]. There are few reports on PK studies of CY and CY metabolite concentrations and post-transplant outcomes in PTCY. In adults, severe chronic GVHD was higher and GRFS was significantly lower in the group with a lower AUC of CEPM [78]. In paediatric PTCY, acute renal and myocardial damage was observed in patients with a higher AUC [79,80]. With regard to CY and CY metabolite concentrations in haplo-HSCT with PTCY, it was expected that there would be marked individual differences in CY metabolite concentrations and, thus, individual differences in the development of complications. However, further large-scale studies are required to confirm these hypotheses.

Finally, this review discusses cardiotoxicity associated with HSCT with PTCY. In a retrospective study of adult patients who underwent HSCT with (*n* = 136) and without (*n* = 195) PTCY, the cumulative incidence of early post-transplant cardiac events was significantly higher in the HSCT with PTCY group (19%) than in the HSCT without PTCY group (6%) [81]. The major cardiac events that occurred after HSCT with PTCY were left ventricular systolic dysfunction (13%), acute pulmonary oedema (7%), pericarditis (4%), arrhythmia (3%), and acute coronary syndrome (2%) [81]. In the multivariate analysis, HSCT with PTCY was associated with cardiac complications [81]. Conversely, a retrospective study of another adult HLA-matched allogeneic HSCT population reported a 7.4% incidence of cardiotoxicity in patients who received PTCY (*n* = 272) compared with 5.8% in patients who did not receive PTCY (*n* = 313), with no significant differences [82]. Factors such as older age, hypertension, arrhythmia, diabetes, and cardiac complications were more predictive of post-transplant cardiotoxicity than PTCY [82]. There have been no case series reports on cardiotoxicity after paediatric HSCT with PTCY. However, it should be noted that even children with no cardiac function or cardiovascular complications before HSCT can have severe complications after PTCY, ranging from pericardial effusion to cardiac tamponade, and may require thoracoscopic pericardioplasty [80].

## 8. Conclusions and Future Perspectives

Haplo-HSCT with PTCY for haematological malignancies has become a safe procedure comparable with HSCT from an HLA-MUD, even in paediatric patients. For haplo-HSCT with PTCY for paediatric haematologic tumours, the conditioning regimen can be MAC or RIC, and the donor source can be BM or PB, depending on the case. Other factors, such as donor selection (sex, age, ABO blood type, and HLA disparity), dose of infused CD34^+^ cells, timing of calcineurin inhibitor initiation, and post-transplant maintenance therapy, can also improve the outcomes of HSCT.

## Figures and Tables

**Table 1:** Previous reports of HLA–haploidentical haematopoietic stem cell transplantation with post-transplant cyclophosphamide for paediatric haematologic malignancies.

Number of Cases	Median Age (Years)	Diagnosis	Disease Status at Transplantation	Conditioning Regimen	Donor Source	GVHD Prophylaxis Other Than PTCY	aGVHD (III–IV) cGVHD (Extensive)	References
15	7	ALL, AML, NB, etc.	non-CR 87%	RIC 100%	BM 100%	CNI, mPSL PTCY (day 3 only)	40% 0%	[28]
23	15 (1–26)	ALL, AML, MDS, etc.	non-CR 26%	MAC 70% RIC 30%	PB 100%	CNI, MMF	5% 12%	[29]
33	12 (1–21)	ALL, AML, lymphoma, etc.	non-CR 30%	MAC 42% RIC 57%	BM 91% PB 9%	CNI, MMF	3% N.D.	[30]
34	11 (0.9–21)	ALL, AML, non-malignant, etc.	CR 68% N/A 32%	MAC 100%	PB 100%	CNI, MMF	5.9% 9.1%	[31]
52	9 (1–17)	ALL, AML, MDS, etc.	non-CR 9.8%	MAC 100%	BM 60% PB 40%	CNI, MTX 81 % CNI, MMF 19 %	8.5% N.D.	[32]
41	6	ALL, AML, MDS, etc.	MRD > 0.01 36%	MAC 100%	BM 22% PB 78%	CNI, MMF	28% N.D.	[33]
42	11 (2–17)	ALL, AML, JMML, etc.	non-CR 3%	MAC 100%	PB 100%	CNI, MMF	17% N.D.	[34]
23	9	ALL, AML	N.D.	RIC 100%	BM or PB	CNI, MMF	N.D. 5.0%	[35]
180	9	ALL	non-CR 19%	MAC 77% RIC 23%	BM 64% PB 36%	CNI, MMF	12.4 % 9.%	[36]

HLA, human leukocyte antigen; GVHD, graft-versus-host disease; ALL, acute lymphoblastic leukaemia; RIC, reduced intensity conditioning; MAC, myeloablative conditioning; PTCY, post-transplant cyclophosphamide; MMF, mycophenolate mofetil; AML, acute myeloid leukaemia; aGVHD, acute graft-versus-host disease; cGVHD, chronic graft-versus-host disease; NB, neuroblastoma; MDS, myelodysplastic syndrome; JMML, juvenile myelomonocytic leukaemia; CR, complete remission; MRD, minimal/measurable residual disease; BM, bone marrow; PB, peripheral blood; CNI, calcineurin inhibitor; mPSL, methylprednisolone; N.A., not applicable; N.D., not dated; MTX, methotrexate.

**Table 2:** Conditioning regimens in haploidentical haematopoietic stem cell transplantation with post-transplant cyclophosphamide at our centre.

MAC	–9	–8	–7	–6	–5	–4	–3	–2	–1	0
TBI-based regimen										
TBI 9.9–12 Gy		↓	↓	↓						
fludarabine 30 mg/m^2^/day					↓	↓	↓	↓		
Non-TBI regimen										
fludarabine 30 mg/m^2^/day		↓	↓	↓	↓					
melphalan 90 mg/m^2^/day						↓	↓			
fludarabine 30 mg/m^2^/day	↓	↓	↓	↓						
targeted busulfan (total AUC 80–100 mg × h/L)					↓	↓	↓	↓		
RIC										
fludarabine 30 mg/m^2^/day		↓	↓	↓	↓					
melphalan 70 mg/m^2^/day						↓	↓	↓		
fludarabine 30 mg/m^2^/day				↓	↓	↓	↓	↓		
cyclophosphamide 14.5 mg/kg/day				↓	↓					
TBI 2 Gy									↓	

MAC, myeloablative conditioning; TBI, total body irradiation; AUC, area under the concentration-time curve; RIC, reduced intensity conditioning; ↓, date of administration or irradiation.
